# Extreme enhancement of superconductivity in epitaxial aluminum near the monolayer limit

**DOI:** 10.1126/sciadv.adf5500

**Published:** 2023-03-01

**Authors:** Werner M. J. van Weerdenburg, Anand Kamlapure, Eirik Holm Fyhn, Xiaochun Huang, Niels P. E. van Mullekom, Manuel Steinbrecher, Peter Krogstrup, Jacob Linder, Alexander Ako Khajetoorians

**Affiliations:** ^1^ Institute for Molecules and Materials, Radboud University, 6525 AJ Nijmegen, Netherlands.; ^2^ Center for Quantum Spintronics, Department of Physics, Norwegian University of Science and Technology, NO-7491 Trondheim, Norway.; ^3^ NNF Quantum Computing Programme, Niels Bohr Institute, University of Copenhagen, 2100 Copenhagen, Denmark.

## Abstract

BCS theory has been widely successful at describing elemental bulk superconductors. Yet, as the length scales of such superconductors approach the atomic limit, dimensionality as well as the environment of the superconductor can lead to drastically different and unpredictable superconducting behavior. Here, we report a threefold enhancement of the superconducting critical temperature and gap size in ultrathin epitaxial Al films on Si(111), when approaching the 2D limit, based on high-resolution scanning tunneling microscopy/spectroscopy (STM/STS) measurements. Using spatially resolved spectroscopy, we characterize the vortex structure in the presence of a strong Zeeman field and find evidence of a paramagnetic Meissner effect originating from odd-frequency pairing contributions. These results illustrate two notable influences of reduced dimensionality on a BCS superconductor and present a platform to study BCS superconductivity in large magnetic fields.

## Teaser

The superconductivity of ultrathin Al enhances threefold and exhibits unexpected vortex behavior in high in-plane magnetic field.

## INTRODUCTION

Bardeen-Cooper-Schrieffer (BCS) theory has been vastly successful at explaining the behavior of conventional superconductors (*1*). Yet, superconductors, both conventional and unconventional, can exhibit complex and unexpected behavior when one or more length scales approach a lower dimensional limit (*2*). While the superconducting critical temperature (*T*_c_) of some materials reduces in the monolayer limit, compared to the bulk (*3*–*5*), it has also been shown that *T*_c_ can be greatly enhanced in this regime, as illustrated by FeSe/SrTiO_3_ (*6*). Likewise, superconductivity can emerge at the interface of two insulating materials, as exemplified by the interface of LaAlO_3_/SrTiO_3_ (*7*). As many types of quantum technologies depend on the growth of superconductors integrated into heterostructures, including superconducting spintronic devices (*8*), high-precision magnetometers (*9*), and qubits based on superconducting nanostructures (*10*), it is imperative to understand what the role of dimensionality and the influence of the environment is on the superconductivity.

Elemental aluminum (Al) is exemplary of a type I BCS superconductor in the weak-coupling regime (*1*) and exhibits unexpected modifications to its superconducting behavior when scaled to the two-dimensional (2D) limit. It has been shown that the critical temperature of Al can be increased from its bulk value of *T*_c_ = 1.2 K by growing thin films, both epitaxial and granular. However, widely varying growth procedures resulting in oxidized films (*11*–*18*), granular Al (*19*–*21*), Al nanowires (*22*, *23*), or doped Al films (*24*, *25*) give dispersing values for *T*_c_ clouding ultimately what contributes to the aforementioned enhancement. In some of these studies, the cleanliness of the interface and the Al itself, as well as the relevant thickness, is ill-defined. Moreover, these studies are often limited to a regime where the thickness is greater than six monolayers (MLs), mainly due to the challenges to synthesize monolayer scale epitaxial Al films. The dispersive findings question to what extent the enhancement of superconductivity is intrinsic to Al itself and to what extent the trend of increasing *T*_c_ persists as films are thinned down further. To this end, experimental approaches that combine high-purity growth methods in a controlled ultrahigh vacuum (UHV) environment with a concurrent in situ characterization are vital to identify the intrinsic superconducting behavior of Al films near the 2D limit. In addition to the observed enhancement of *T*_C_, the upper critical field in the direction parallel to the film surface has been shown to increase substantially (*16*). Because of the low spin-orbit scattering rate in Al, these films characteristically show the Meservey-Tedrow-Fulde (MTF) effect, where the application of a magnetic field gives rise to a spin splitting of the quasiparticle excitations (*26*, *27*). In addition, it has been proposed that this high-field regime can promote odd-frequency spin-triplet correlations (*28*–*32*), but it has been challenging to confirm their presence experimentally (*28*, *33*, *34*). The combination of thin film Al and large magnetic fields, as used in superconducting qubit devices, especially those aiming to induce topological superconductivity (*10*, *35*, *36*), puts forward questions about how superconductivity is affected by external magnetic fields and the role of unconventional pairing.

Here, we show that Al(111) films epitaxially grown on Si(111)–(7 × 7), approaching the monolayer limit, exhibit a greatly enhanced *T*_c_, up to about a factor of three, when compared to the bulk value. Using scanning tunneling microscopy/spectroscopy (STM/STS) at variable temperatures down to millikelvin, we first characterize the structural and large-scale electronic properties of epitaxial films of Al grown on Si(111) for various thicknesses (*N*). We subsequently characterize the associated superconducting gap (Δ) with each grown film. For the largest gap values, we corroborate these measurements with *T*_c_ by measuring Δ(*T*). Next, we probe the magnetic field–dependent properties of individual Al films for different thicknesses in magnetic fields with different field orientations. We confirm the expected type II behavior in out-of-plane magnetic fields, including the observation of an Abrikosov lattice. For in-plane magnetic fields, we observe the MTF effect and use the spectral evolution in magnetic field to quantify the *g*-factor of the various films, which are all shown to exhibit *g* ≈ 2. We finally characterize the vortex structure in the presence of the MTF effect, which shows a reshaping of the vortex structure when compared to zero in-plane field. Based on numerical simulations using the Usadel equation, we quantify the observed structure and relate it to the presence of both even and odd-frequency pairing correlations as well as their contribution to the screening currents.

## RESULTS

### Structural and spectroscopic properties of epitaxial Al films

**Fig. 1.**
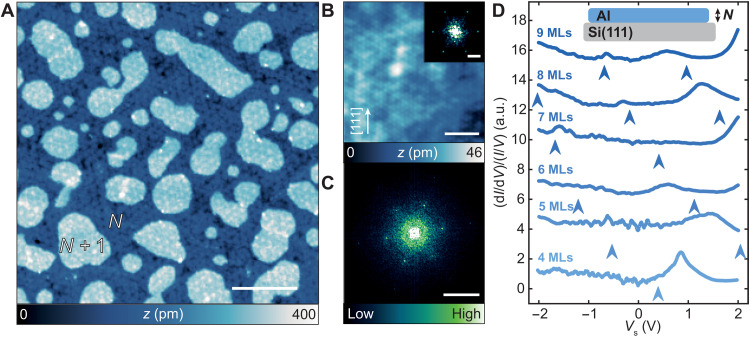
Structural and spectroscopic properties of ultrathin epitaxial Al films. (**A**) Constant-current STM image of an Al/Si(111) sample with 8.5-ML coverage (*V*_s_ = 100 mV, *I*_t_ = 20 pA; scale bar, 20 nm). (**B**) Constant-current STM image with atomic resolution (coverage: 35.1 MLs, *V*_s_ = 3 mV, *I*_t_ = 500 pA; scale bar, 2 nm). Inset: fast Fourier transform (FFT) of the image in (B) (scale bar, 2 nm^−1^). (**C**) FFT of the image in (A) (scale bar, 0.5 nm^−1^). (**D**) Spectroscopy taken on 4- to 9-ML layers (stabilized at *V*_s_ = 2 V, *I*_t_ = 200 pA, *V*_mod_ = 5 mV, *T* = 1.3 K). The d*I*/d*V* signal (in arbitrary units; a.u.) is normalized by *I*/*V* to correct for the transmission factor of the tunneling barrier. Arrows indicate the calculated QWS energies from density functional theory in (*42*) (see section S1 and fig. S3). Inset sketch shows an Al film on Si(111) with a thickness of *N* MLs.

Epitaxially grown Al films (see Materials and Methods) imaged with STM typically show a closed film of a given thickness, decorated with a density of islands a monolayer higher (Fig. 1A and fig. S2). Films with a given thickness exhibit two different periodicities (Fig. 1, B and C). A short-range threefold periodicity with *a* ≈ 0.25 nm coincides with the expected atomic lattice constant of Al(111). In addition to the atomic periodicity, a long-range periodicity can be observed in films for thicknesses up to 26 MLs, which is also threefold symmetric and exhibits a periodicity *a*_M_ ≈ 2.6 nm. This periodicity is commensurate with the underlying 7 × 7 reconstruction of Si(111) (*37*, *38*), and it is reminiscent of the moiré-type structures seen for other thin superconducting films (*39*, *40*). The appearance of both the moiré-type structure and the atomic periodicity is indicative that the interface is most likely pristine with negligible intermixing at the growth temperatures used. Epitaxial film growth is observed for Al films ≥4 MLs, as identified in (*38*). In attempts to measure even thinner Al films, our growth procedure resulted in broken and granular films.

The thickness of a given film can be corroborated with STS measured in a voltage range of ±2 V. For a given *N*, layer-dependent broad peaks can be identified at given voltages, which vary depending on the given value of *N* (Fig. 1D). To better illustrate the measured peaks for both filled and empty states, d*I*/d*V* spectroscopy was normalized to *I*/*V*. Moreover, different films with the same value of *N* reproducibly show the same spectroscopic features, enabling spectroscopic fingerprinting of the layer thickness, although the films are closed (see section S1 and fig. S3). The appearance of such peaks in STS is reminiscent of quantum well states (QWS) typically observed on other thin metallic films grown on Si(111) (*41*). For reference, the QWS energies extracted from (*42*, *43*) are indicated in Fig. 1D by blue arrows underneath each measured spectrum. In this comparison, the QWS energies do not exactly match the measured peak positions, but there is a qualitative agreement between the energy difference between adjacent QWS, and the measured spectra, up to approximately 13 MLs. As seen from previous angle-resolved photoemission spectroscopy (ARPES) measurements (*44*) and the aforementioned calculations, the expected QWS have a smaller effective mass and are expected to disperse, when compared to the QWS of Pb/Si(111) (*41*). This inherently weakens the QWS intensity and makes a direct mapping of the exact QWS onset energies based solely on point-STS measurements imprecise. We note that a direct comparison to measured ARPES (*44*) is challenging, as we observe stronger features in the empty state region of the spectra, where there are no ARPES measurements. Likewise, ARPES spatially averages over regions of the film where we expect spectroscopic contributions from multiple thicknesses of the film.

### Superconducting gap and critical temperature as a function of film coverage

**Fig. 2.**
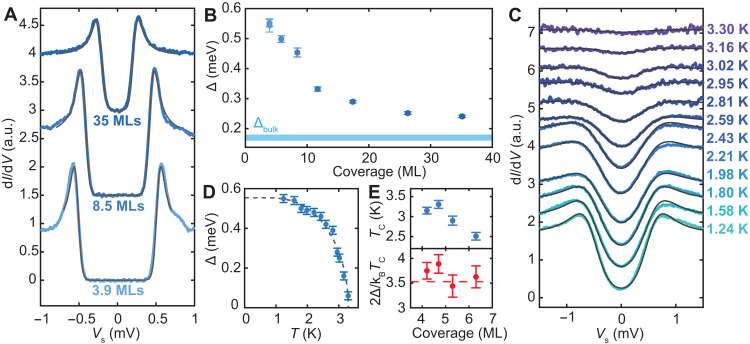
Δ and *T*_c_ enhancement for ultrathin Al films. (**A**) Superconducting gap spectra taken at *T* = 30 mK for samples with varying Al coverage (artificially offset) with Maki fits (gray lines) (stabilized at *V*_s_ = 3 mV, *I*_t_ = 200 pA, *V*_mod_ = 20 μV). (**B**) Extracted Δ at *T* = 30 mK for varying Al coverage, where the error bar represents the SD of Δ in an ensemble of 18 to 30 spectra. (**C**) Temperature-dependent superconducting gap spectra (artificially offset) for 4.5-ML coverage, manually matched with the Dynes equation (gray lines) (stabilized at *V*_s_ = 5 mV, *I*_t_ = 300 pA, *V*_mod_ = 100 μV). (**D**) Extracted Δ as a function of temperature with the BCS fit with *T*_c_ = 3.3 ± 0.1 K and Δ^*T =* 0^ = 0.55 ± 0.02 meV. (**E**) Extracted *T*_c_ values and 2Δ/*k*_B_*T*_c_ ratios for four Al coverages (see fig. S7; error bars represent SD from the BCS fit). We note that the films in (C) to (E) were not measured at *T* = 30 mK and are therefore excluded from (B).

We measured Δ as a function of coverage using high-energy resolution STS at variable temperature. Here, the coverage of a given film is defined as the cumulative Al material of its main layer and (vacancy) islands. Below, we first detail the spectral gap as measured at the lowest temperature, namely, *T* = 30 mK, for three coverages in Fig. 2A. A typical spectrum shows a BCS-like, hard gap structure symmetric around *V*_s_ = 0 mV and sharp coherence peaks at the gap energy Δ, which can be fitted and extracted with a broadened Maki function (see section S2 and fig. S5 for a discussion on the possible broadening contributions) (*45*). We find that the gap value shows the largest enhancement of Δ = 0.560 ± 0.015 meV for a coverage of 3.9 MLs (4 MLs with a distribution of vacancy islands), which is more than a threefold enhancement compared to the bulk value of Δ_bulk_ = 0.16 to 0.18 meV (*46*, *47*). We find that the spectra taken at various locations on the sample, including on (vacancy) islands and along the long-range periodicity, reveal a uniform superconducting gap with a constant Δ (± 0.02 meV) and small variations in coherence peak height (see fig. S4). Therefore, we assign Δ for each sample as the spatial average of all gap values extracted from ≥18 spectra, where the error bar represents the standard deviation of those values. The uniformity in the value of Δ is in contrast to the variations in the band structure on larger energy scales, where we see clear differences in STS for different layer heights. This observation suggests that the value of Δ is not significantly modulated due to the presence of different QWS stemming from variations in the film thickness, in contrast to reports on Pb/Si(111) (*39*, *48*) and in line with observations for Pb/BP (*49*).

Measurements on films with different coverage yield a monotonously increasing trend in Δ as the film coverage is lowered, as shown in Fig. 2B for samples between 4 and 35 MLs. Here, each data point represents one grown sample. For the largest coverages we measured, namely, 35 MLs, we still observed a slight enhancement in Δ compared to the bulk value (blue bar), as was also seen in (*18*). The monotonous trend contrasts the observations for Pb/Si(111), where the critical temperature oscillates due to a modulation of the local density of states (LDOS) at *E*_F_. Here, we see no clear correlation between the QWS energies and the corresponding gap size.

To quantify *T*_c_ in relation to the measured values of Δ at millikelvin temperature, we performed temperature-dependent measurements of Δ*(T*) for four different film coverages (see Materials and Methods for details and section S3 and fig. S6 for the temperature calibration). Δ(*T*) was measured for a given sample by incrementally raising the sample temperature between 1.3 and 4.0 K. With increasing *T*, Δ(*T*) shows the expected decrease until the gap is eventually fully quenched, coinciding with *T*_c_ (Fig. 2C). To quantify the value to *T*_c_, we first fitted each measured spectra with a BCS Dynes function (see section S2) (*50*). We subsequently fitted the numerically determined temperature dependence of the gap within BCS theory to the extracted Δ(*T*), as exemplified for an Al film with a 4.7-ML coverage in Fig. 2D, and find *T*_c_ = 3.31 ± 0.11 K. In Fig. 2E, we illustrate the extracted *T*_c_ for four different films (see fig. S7). Based on BCS theory, the ratio between *T*_c_ and Δ(*T =* 0) leads to an expected ratio of 2Δ(*T* = 0)/*k*_B_*T*_c_ = 3.53, which typically describes superconductors in the weak-coupling limit, such as bulk Al (*46*, *47*). Based on the extracted values, we plot the ratio between Δ and *T*_c_ in Fig. 2E. The overall trend indicates that the ratio is in close agreement to the expected value 3.53 as seen for the bulk Al, suggesting that the thin Al films studied here may be in the weak-coupling limit. We note that the *T*_c_ was only measured for four films, and not for a given film multiple times. Therefore, the error bars coincide with the standard deviation given by the fits shown in Fig. 2D and fig. S7. To infer a coverage-dependent trend in the extracted ratio, further measurements are needed. Moreover, the effect of the sample morphology and defects on the gap value and the ratio requires further study.

The threefold enhancement of Δ and *T*_c_ is distinctly larger than reported epitaxial Al films in the literature, where capped films were studied ex situ only down to 6 MLs (*18*). Likewise, it exceeds most reported values for *T*_c_ of other studies on oxidized (single) Al films (*12*–*18*, *20*, *21*), likely due to the thinner films, the crystallinity, and the absence of the oxide layer. This observation directly refutes an early idea that the origin of the enhancement effect was due to the oxygen layer (*12*). In other reports (*24*, *25*), enhanced values of *T*_c_ for Al films were obtained by doping with ~2% of Si impurities. However, potential intermixing of Si and Al with this quantity of impurities would likely obscure the moiré pattern and atomic-resolution images presented in Fig. 1. In addition, we can also exclude a considerable influence of Si intermixing on the enhancement of superconductivity, since we do not observe a considerable change in gap enhancement for films when the annealing time (and thus potential intermixing) is minimized (see section S4 and fig. S8). These observations indicate that the enhanced superconductivity is an intrinsic property of ultrathin Al films, but it remains an open question if other weak-coupling superconductors present similar enhancement effects and what the role of the substrate/interface is (*4*).

### Abrikosov lattice and out-of-plane magnetic field response

**Fig. 3.**
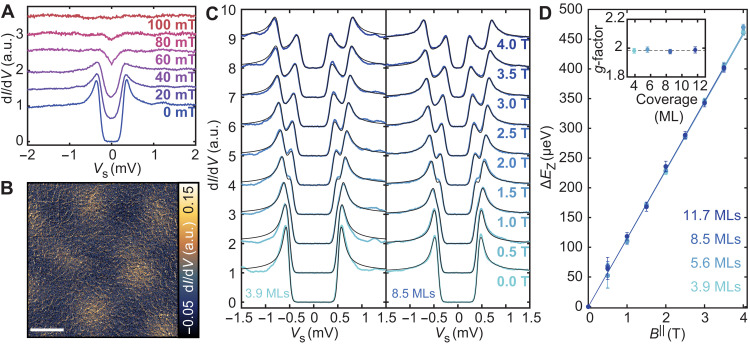
Magnetic field response of Al films. (**A**) Evolution of the superconducting gap in out-of-plane magnetic field *B*^⊥^ for an 11.7-ML film, measured in between vortices in (B). (**B**) Constant-contour d*I*/d*V* map with *B*^⊥^ = 50 mT (height profile recorded at *V*_s_ = 10 mV, *I*_t_ = 10 pA; image taken with *V*_s_ = 0 mV, *V*_mod_ = 20 μV; scale bar, 100 nm). (**C**) Evolution of the superconducting gap as a function of in-plane magnetic field *B*^∥^ for a 3.9- and 8.5-ML film. Black lines are fits using two Maki functions separated by Zeeman splitting Δ*E*_z_ (stabilized at *V*_s_ = 3 mV, *I*_t_ = 200 pA, *V*_mod_ = 20 μV). (**D**) Extracted Δ*E*_z_ for four Al coverages (see fig. S10). The solid lines are weighted linear fits to extract the *g*-factor for each sample (inset).

Subsequently, we characterize the magnetic field–dependent response of various Al films in two magnetic field orientations, i.e., perpendicular/parallel to the surface. First, we quantify the upper critical field for an Al film with an 11.7-ML coverage in a magnetic field perpendicular to the film plane ($B_{c2}^{⊥}$). By incrementally increasing *B*^⊥^ and measuring local point spectra (Fig. 3A), the coherence peaks flatten and the zero-bias conductance increases gradually until the gap has completely vanished at *B*^⊥^ = 100 mT. This upper limit for $B_{c2}^{⊥}$ gives an estimate for the coherence length ξ of ~64 nm, as $\xi =\sqrt{{\text{Φ}}_{0}/2\pi B_{c2}^{⊥}(T=0)}$, where Φ_0_ is the magnetic flux quantum (*51*). The expected type II behavior can be observed by spatially imaging the zero-bias conductance for nonzero values of *B*^⊥^. We measured a constant-contour d*I*/d*V* conductance map at *V*_s_ = 0 mV (*B*^⊥^ = 50 mT), which reveals an Abrikosov lattice, with a vortex radius on the order of the coherence length (Fig. 3B and fig. S9).

### MTF effect and the Clogston-Chandrasekhar limit

After characterizing the out-of-plane response, we characterized the response of various films to an in-plane magnetic field (*B*^∥^) for various coverages. Since screening currents cannot build up in the confined superconductor, orbital depairing is absent, and the magnetic field penetrates the superconductor, allowing us to study the superconducting state in combination with large magnetic fields compared to the typical out-of-plane critical values. In the absence of spin-orbit scattering, the quasi-particle excitations of the superconductor are sufficiently long-lived to observe the MTF effect in this regime (*26*, *27*). This effect is exemplified by a spin-splitting of the coherence peaks, where each peak shifts by ± *g*μ_B_*SB*^∥^, giving a total Zeeman splitting of ∣*E_z_*∣ = *g*μ_B_*B*^∥^ for *S* = 1/2. For a homogeneous superconductor in the absence of spin-orbit coupling, the superconducting state may only persist up to the Clogston-Chandrasekhar limit (*52*, *53*), given by $h=\text{Δ}/\sqrt{2}$, with *h* = μ_B_*B*^∥^, where a first-order phase transition to the normal state occurs.

In Fig. 3C, we illustrate the measured MTF effect for two Al films with a coverage of 3.9 and 8.5 MLs, where the STS was measured for increasing values of *B*^∥^, up to *B*^∥^ = 4 T. The manifestation of the MTF effect is the appearance of a spin-split gap structure. We quantify the splitting in Fig. 3C by subdividing the gap structure into two independent spin-polarized distributions and fitting two Maki functions with equal gaps, shifted with respect to each other by the Zeeman energy Δ*E*_z_. As illustrated in Fig. 3D, we measured Δ*E*_z_(*B*^‖^) for four film coverages (also see fig. S10) and quantified the splitting of the coherence peaks at each field increment. The resulting linear trend is used to extract the *g*-factors (see inset of Fig. 3D) with an average of *g* = 1.98 ± 0.02 (where *g* = Δ*E*_z_/μ_B_*B*^∥^ for *S* = 1/2). This measurement shows that the quasiparticles in the ultrathin regime remain free-electron like, and the linearity of the graph further illustrates that spin-orbit coupling is negligible in these films. In addition, we note that the expected Clogston-Chandrasekhar limit for the 8.5-ML film is at $B_{\mathrm{CC}}^{∥}=\text{Δ}/\sqrt{2}\mu_{B}∼5.5\;T$, i.e., above our experimental limit of *B*^∥^ = 4.0 T. However, for films with a smaller gap size (with coverages of 11.7 and 17.4 MLs), we could observe a sudden quenching of superconductivity at in-plane fields near the theoretical limit.

### Vortex structure in the presence of the MTF effect

**Fig. 4.**
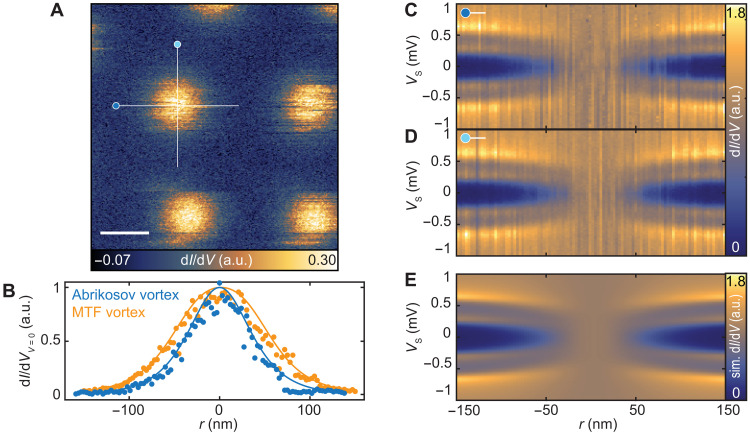
MTF vortex in vector magnetic field. (**A**) Constant-contour d*I*/d*V* map in vector magnetic field with *B*^∥^ = 2.99 T and *B*^⊥^ = 30 mT for an 8.5-ML film (height recorded at *V*_s_ = 10 mV, *I*_t_ = 10 pA; image taken with *V*_s_ = 0 mV and *z* offset = 100 pm, *V*_mod_ = 50 μV; scale bar, 100 nm). (**B**) Vortex profile at *V*_s_ = 0 mV, extracted from the line spectra across the Abrikosov vortex (blue; see fig. S9, B and C) and the MTF vortex (orange; C and E). Both the experimental (scatter points) and theoretical (lines) zero-bias conductance profiles are presented. (**C** and **D**) d*I*/d*V* spectra along a horizontal and vertical line across a vortex core (stabilized at *V*_s_ = 3 mV, *I*_t_ = 200 pA, *V*_mod_ = 20 μV). (**E**) Simulated d*I*/d*V* signal by solving the self-consistent gap equation (see section S5) using *h*^∥^/Δ^∞^ = 0.38, ξ = 42 nm, Γ = 0.007 Δ^∞^, κ = 5 and broadened with *T*_eff_ = 250 mK.

The manifestation of the MTF effect in ultrathin Al films provides an opportunity to explore the atomic-scale variations in the conductance in response to variable magnetic field, for example, the resultant vortex behavior in the presence of the MTF effect. Moreover, the presence of large in-plane magnetic fields can induce pairing contributions in the form of odd-frequency spin-triplet correlations, which may act differently around a vortex and exhibit a paramagnetic Meissner response (*33*, *54*, *55*). Using a vector magnetic field, we induced vortices in a given Al film with *B*^⊥^ = 30 mT and simultaneously applied *B*^∥^ = 2.99 T to enter the MTF regime. We subsequently spatially mapped the zero-bias conductance in constant-contour mode, as illustrated for an 8.5-ML Al film (Fig. 4A). The resulting image shows multiple round vortices with an expected flux density (see also section S5). Note that the vortices may occasionally move, likely due to interactions with the tip (also see figs. S9 and S11). This can yield vortices that appear noisy as well as obscure the symmetry of underlying vortex lattice. To further characterize the structure, we also performed STS along a horizontal and vertical line across a given vortex (Fig. 4, C and D). Both directions show a split gap structure with Δ = 0.45 meV at ~150 nm from the vortex center and a gradual decrease of Δ toward the center with a constant Zeeman splitting. Closer to the vortex center, the spectral gap is rapidly quenched, resulting in an extended region of ~70 nm in diameter without any spectroscopic indications of superconductivity. In this regime, the apparent region with conductance associated with the normal state is radially larger than what is expected for a typical vortex in the absence of an in-plane magnetic field component (e.g., fig. S9). Besides this extended region where the quasiparticle gap is zero, the total radius of a vortex in the MTF regime is also larger compared to the typical vortex shape in the absence of an in-plane magnetic field, as illustrated by comparing the zero-bias conductance profiles in Fig. 4B (also see fig. S9).

To explain the observation of the vortex structure in the presence of the MTF effect, or the MTF vortex for short, we modeled the superconducting vortex structure using the quasiclassical Keldysh Green’s function formalism (*56*, *57*), assuming a single-phase winding in the superconducting gap parameter. We assume that the coherence length of the superconductor is large compared to the mean free path, dictated by the sample morphology (sample thickness, island size, and moiré periodicity), such that the quasiclassical Green’s function solves the Usadel equation (*58*). Therefore, we consider the diffusive limit, where only *s*-wave correlations can persist. This is in contrast to considerations in the ballistic limit (*31*). We fix Δ^∞^, the gap size at infinite distance from the vortex, and the spin-splitting field *h*^∥^ = μ_B_*B*^‖^ to the experimental values (*h*^∥^/Δ^∞^ = 0.38) and solve the Usadel equation self-consistently with both the superconducting gap equation and Maxwell’s equations (see section S5 for more details). In Fig. 4E, we illustrate the calculated density of states and account for Dynes broadening as well as experimental broadening by convoluting with the Fermi-Dirac distribution with *T*_eff_ = 250 mK. The simulated distance-dependent spectra show an excellent agreement with the experimental data, reproducing the zero-bias conductance profiles (Fig. 4B), the evolution of the spin-split gap structure, and the extended region with a quenched quasiparticle gap (see fig. S9 for the calculated profile for *h*^∥^/Δ^∞^ = 0). In addition, we can extract the coherence length of ξ = 42 nm.

**Fig. 5.**
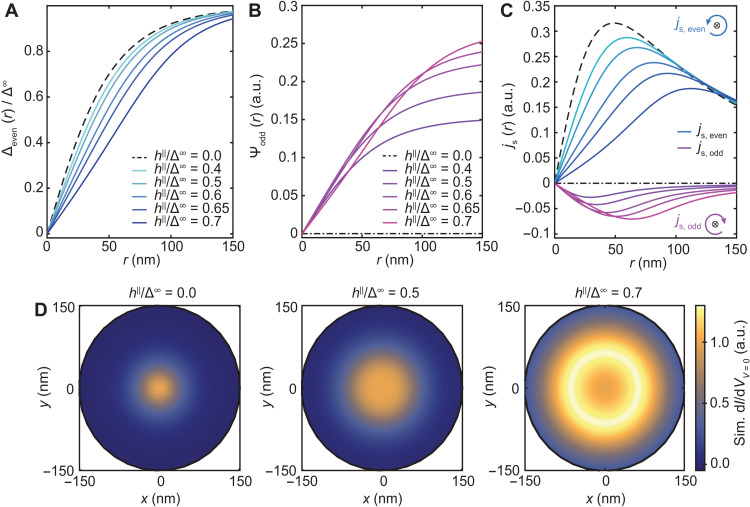
Evolution of the MTF vortex structure with in-plane magnetic fields. Calculated gap function across a vortex for (**A**) ω_e_ spin-singlet pairs, (**B**) ω_o_ spin-triplet pairs, and (**C**) the calculated electric supercurrent density for various in-plane magnetic fields [color used consistently in (A) to (C)]. The supercurrent flow around the magnetic flux line (⊗) is indicated schematically. (**D**) Simulated zero-bias conductance represented spatially for three *h*^∥^/Δ^∞^ ratios (ξ = 42 nm, Γ = 0.001 Δ^∞^, κ = 5, as defined in section S5).

The theoretical model provides a detailed understanding of the MTF vortex structure in a varying in-plane magnetic field. First, the solution to the gap equation consists of both even-frequency (ω_e_) spin-singlet $\frac{1}{\sqrt{2}}$(|↑↓⟩ − |↓↑⟩) and odd-frequency (ω_o_) spin-triplet $\frac{1}{\sqrt{2}}$(|↑↓⟩ + |↓↑⟩) pairing contributions. Therefore, there is always a coexistence of both types of pairing contributions in the presence of an in-plane magnetic field. To understand the vortex structure, it is important to identify the role of both types of pairing contributions. In Fig. 5 (A and B), we plot the contributions of ω_e_ and ω_o_ pairing correlations, Δ_even_ and Ψ_odd_, respectively, as a function of distance across the MTF vortex structure, where *r* = 0 refers to the vortex center. Toward the vortex core, both order parameters decrease monotonically and gradually as the distance to the core is reduced. By evaluating the gap equation for increasing values of *h*^∥^, we find an increasing contribution of ω_o_ pairs, as well as a more extended and gradual vortex profile. The combination of the shallow vortex shape and the presence of ω_o_ correlations near the vortex core, which are more susceptible to single-particle excitations (*54*), explains the extended quenched gap region, despite a finite order parameter being present in this region. We also note that close to the vortex center, Δ_even_ is reduced beyond the Clogston-Chandrasekhar limit, which is only allowed for a local region in the superconductor.

Mesoscopically, the presence of vortices is driven by a circulating supercurrent that screens the penetrating magnetic flux. Therefore, we additionally calculated the ω_e_ and ω_o_ contributions to the supercurrent density and plot this as a function of distance in Fig. 5C for various values of *h*^∥^/Δ^∞^. In the absence of *h*^∥^, we find the characteristic diamagnetic response of the screening current (*59*) (black dashed lines), consisting of purely ω_e_ pairs. At finite values for *h*^∥^/Δ^∞^, we find two contributions to the screening current with opposite signs, originating from the ω_e_ and ω_o_ pairing correlations. This demonstrates a paramagnetic Meissner contribution from the ω_o_ pair correlations. With increasing *h*^∥^/Δ^∞^, both screening current contributions extend further outward, and the paramagnetic component increases in amplitude, but the total screening current (i.e., the sum of both contributions) remains diamagnetic. In this way, the paramagnetic contribution to the supercurrent, originating from the odd-frequency correlations induced in the MTF regime, gives rise to an enhanced magnetic penetration depth and contributes to the enhanced vortex size.

In addition to the aforementioned details, we calculated how the measurable vortex structure evolves as a function of *h*^∥^. Figure 5D provides a visual representation of the simulated spatial d*I*/d*V* signal at *V*_s_ = 0 mV, showing the evolution of the vortex structure. For *h*^∥^/Δ^∞^ = 0, the vortex starts as the expected structure with a sharp rise in conductance at the core (also see fig. S9). For a persistently rising field value, the high-conductance region broadens and flattens out near the core, as can be seen for *h*^∥^/Δ^∞^ = 0.5, and finally develops a high-intensity ring around the vortex core at *h*^∥^/Δ^∞^ = 0.7 due to the overlap of pronounced inner coherence peaks.

**Fig. 6.**
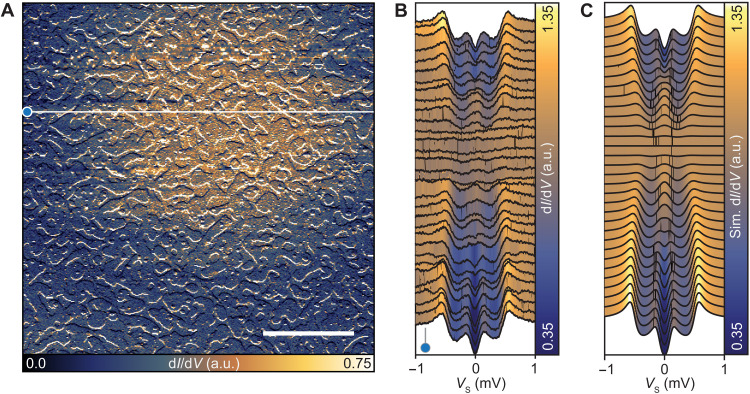
Zero-bias peak in the MTF vortex profile for large *h^∥^/*Δ*^∞^*. (**A**) Constant-contour d*I*/d*V* map with *B*^∥^ = 3.6 T (*B*^⊥^ = 0 mT) for an 11.7-ML film (height recorded at *V*_s_ = 10 mV, *I*_t_ = 10 pA; image taken with *V*_s_ = 0 mV and *z* offset = 120 pm, *V*_mod_ = 50 μV; scale bar, 100 nm). (**B**) Spectra measured along a line of 400 nm across a vortex structure [see white line in (A); stabilized at each point with *V*_s_ = 3 mV, *I*_t_ = 200 pA, *V*_mod_ = 20 μV]. (**C**) Simulated d*I*/d*V* signal across a vortex core by solving the self-consistent gap equation using *h*^∥^/Δ^∞^ = 0.63, Γ = 0.1Δ^∞^, κ = 5, ξ = 50 nm and broadened with *T*_eff_ = 250 mK.

We propose that these MTF vortices can appear in any type II superconductor in the presence of a large magnetic field, given that spin-orbit scattering and orbital depairing are negligible. These reshaped vortices are likely to occur in experimental setups, even in the absence of an applied out-of-plane field, since a small misalignment between the sample plane and the in-plane magnetic field direction can induce an out-of-plane component (where $B_{c1}^{⊥}$/$B_{c2}^{\|}$ ≪ 1). In our case, we find a small tilt angle of 0.2° (see section S5 and fig. S11), estimated by the observed vortex density at *B*^∥^ = 4.0 T. Consequently, it is interesting to explore larger ratios of *h*^∥^/Δ^∞^, close to the Clogston-Chandrasekhar limit. In Fig. 6A, we show one instance of a vortex where *B*^∥^ = 3.60 T, while *B*^⊥^ = 0.0 T for an 11.7-ML Al film. Here, STS measurements along a horizontal line and the simulated d*I*/d*V* signal (Fig. 6, B and C) reveal the appearance of a zero-bias peak at finite distance from the vortex core, owing to the gradual merging of the two inner coherence peaks. We expect that for even larger *h*^∥^/Δ^∞^ ratios, this will give rise to a pronounced ring as seen in Fig. 5D. For these films, where $B_{c1}^{⊥}$/$B_{c2}^{\|}$ is very small, small angular offsets in the magnetic field can lead to vortex formation near the Clogston-Chandrasekhar limit. For experiments where large in-plane magnetic fields are needed to induce a topological superconducting phase, the appearance of the aforementioned in-gap states at zero energy may make it more complicated to assign a topological character in this field regime.

## DISCUSSION

In conclusion, we have demonstrated that the superconducting gap size and critical temperature of Al can be enhanced up to threefold in the 2D limit, for films as thin as 4 MLs. Based on thickness-dependent measurements of the superconducting gap combined with variable temperature measurements, we establish that the ratio of Δ to *T*_c_ remains near the expected BCS ratio. While the enhancement of superconductivity can be seen gradually as films reach the 2D limit, it remains an open question how the enhanced superconductivity arises. More specifically, it remains to be explored if, besides electron-phonon coupling, other low-energy excitations become relevant in the lower dimensional limit, such as plasmons. It is also particularly interesting to explore if this enhancement is unique to Al, or if it can be generalized to other superconductors in the weak-coupling limit. In addition to the enhancement of the critical temperature, we quantify the type II behavior of these films, including a characterization of the vortex lattice in the presence of the MTF effect. Notably, we find that the shape of the vortex structure in the presence of the MTF effect is strongly modified, including an experimental observation of a gapless region. Our simulations confirm a connection between the extended vortex shape and the presence of odd-frequency pairing contributions, as exemplified by a paramagnetic contribution to the screening supercurrent. In addition, these results highlight that the presence of pairing correlations and the observation of a tunneling gap are not synonymous in a tunneling experiment (*60*). Therefore, further investigation with pair-sensitive tunneling techniques can provide more insight into the unconventional pairing contributions in the high-field regime of superconductivity (*59*, *61*, *62*).

## MATERIALS AND METHODS

All presented STM/STS measurements were performed using two different homebuilt systems with base temperatures of 30 mK (*63*) and 1.3 K (system A and system B, respectively). All presented experimental data were measured at *T* ≈ 30 mK, unless specified otherwise. Since both systems have an identical UHV chamber design (<5 × 10^−10^ mbar), the sample growth was performed using the same procedures. First, the Si(111) wafer (As doped, resistivity <0.005 ohm·cm) is annealed at ~750°C for >3 hours for degassing purposes by applying a direct current through the wafer. The temperature is measured by aligning a pyrometer onto the wafer surface. Afterward, the Si(111)–7 × 7 reconstruction is prepared by repeated flash-annealing to *T* = 1500 to 1530°C. Second, the Si substrate is cooled on a liquid nitrogen cold stage (~110 K) for low-temperature Al growth. We deposited Al from a crucible with a cold-lip effusion cell (CLC-ST, CreaTec) at an evaporation temperature of *T* = 1030°C, yielding a deposition rate of 0.39 MLs (A) or 1.06 MLs (B) per minute (see section S1 and fig. S1). Third, after depositing the desired amount of material, the sample is placed onto a manipulator arm and annealed at room temperature for 30 min for coverages of >4 MLs and 10 to 20 min for coverages of <4 MLs (A) and 15 min for coverages of 4 to 6 MLs (B). The anneal time is stopped by placing the sample into a flow cryostat–cooled manipulator arm (for system A) and transferring the sample into the STM body.

All samples were measured using an electrochemically etched W tip, which was prepared by dipping into an Au(111) crystal and subsequently characterized. STS measurements were done with a standard lock-in technique, where a sinusoidal modulation voltage (*f*_mod_ = 877 to 927 Hz and *V*_mod_ as indicated in the figure captions) was added to *V*_s_. For variable temperature measurements on system B, we calibrated the used temperature sensor by measuring and fitting the temperature-dependent superconducting gaps of a film of Sn/Si(111) and bulk V(111) (see section S3 and fig. S6).

For vortex imaging, we spatially mapped the d*I*/d*V* signal in constant-contour mode, as done in (*49*). In this mode, we first recorded a constant-current line scan trace, measuring the values of *z*, with a closed feedback loop, at a bias voltage (*V*_s_ = 3 mV). Next, the recorded values of *z* (including a *z* offset) were used at the measuring bias (*V*_s_ = 0 mV). This method was repeated for every line of the image. Sharp topographic features, such as island edges, are likely to contribute to the signal in this measurement mode. In all presented vortex maps, the orientation of the in-plane magnetic field is 10° off the vertical (*y*) axis of the images (*64*–*73*).

## Supplementary Materials

### This PDF file includes:

Sections S1 to S5 Figs. S1 to S11
