# Supplementary material for: Extreme enhancement of superconductivity in epitaxial aluminum near the monolayer limit
Source: Sci Adv. 2023 Mar 1;9(9):eadf5500. doi: 10.1126/sciadv.adf5500 (PMC9977180; doi:10.1126/sciadv.adf5500)
Supplement: Supplementary file 1 — Sections S1 to S5 Figs. S1 to S11 [file sciadv.adf5500_sm.pdf]

Science Advances
AAAS

Supplementary Materials for

Extreme enhancement of superconductivity in epitaxial aluminum near the monolayer limit

Werner M.J. van Weerdenburg *et al.*

Corresponding author: Alexander Ako Khajetoorians, a.khajetoorians@science.ru.nl

Sci. Adv. **9**, eadf5500 (2023)
DOI: 10.1126/sciadv.adf5500

This PDF file includes:

Sections S1 to S5
Figs. S1 to S11

S1 Film morphology and characterization

Calibration of the coverage

Prior to sample growth on Si(111), the deposition rate at T = 1030 °C is characterized by depositing Al onto a quartz microbalance (QMB). We repeat this calibration between multiple sample preparations to ensure that the deposition rate does not substantially change over time. We also calibrated the Al deposition by evaporating Al onto a clean Si(111)-7x7 surface at room temperature, for both STMs used in this study. This growth method yields small islands of thickness ≥ 4 ML, which enable calibration as also seen in ref. (*38*). Fig. S1A shows a constant-current STM image illustrating this calibration, after depositing Al for 12 minutes, followed by an additional 10 minutes waiting time before low temperature characterization. The corresponding apparent height histogram in Fig. S1B shows the density for each specified thickness. Based on this analysis, we extracted a deposition rate of 0.39 ML/min for system A. This rate is used to estimate the coverage of each sample in combination with morphology analysis on large-scale images to precisely determine the amount of main layer and (vacancy) islands.

Film morphology for different coverages

The morphology of the Al films measured in this work vary as a function of coverage and annealing conditions. In Fig. S2, we present the morphology of three different Al films with different coverage, grown with the described sample preparation (see Materials and Methods) and with a room-temperature anneal time of (A) 10 minutes, and (B,C) 30 minutes. The key differences are: (i) the roughness of the film increases as we approach the 4 ML coverage limit, where we still observe a closed, metallic film (ii) the apparent height variation changes for thicker films, e.g. as shown for 35 ML, where samples can show *N*-2/ *N*+2 variations in addition to the *N*-1/*N*+1 variations observed in thinner films. We note that the morphology is extremely sensitive to the annealing time (also see Fig. S7A). However, the length scale related to the mean surface roughness typically remains smaller than the coherence length of the superconductor (i.e. < 50 nm).

Large-scale scanning tunneling spectroscopy and comparison to DFT and ARPES

We measured dI/dV in a large bias voltage range between V_s = ± 2 V on various thicknesses for a given sample. To compare to previous results, we plotted the calculated quantum well state (QWS) energies for Al(111) from DFT calculations derived from refs. (*42, 43*) in the upper panel of Fig. S3A, as well as the extracted QWS energies from ARPES measurements on various Al(111) films derived from ref. (*44*). We note that ARPES macroscopically averages over large areas of the film, compared to STS, and therefore the measured spectra will presumably convolute all layer thicknesses present on the film. Furthermore, we compare the QWS energies from DFT with the peak positions observed in the large-scale STS measurements in the lower panel of Fig. S3A. The spectra for layer thicknesses between 11 ML and 37 ML are shown in Fig. S3B-E.

Spatial measurement of superconductivity

To check the effect of the film morphology (i.e. islands, moiré-type pattern) on the superconductivity, we measured dI/dV spectra along a line across an Al film with 8.5 ML coverage. Fig. S4A shows the main layer (8 ML) and an island (9 ML), as well as the long-range periodicity, with the measured line running across all these morphological features. The spectra along this line (Fig. S4B) show a constant gap size with small variations in the coherence peak height. Similarly, we measured spectra on arbitrary locations on all samples and find negligible variations in the gap size, as indicated by the error bars in Fig. 2B.

S2 Fitting superconducting spectra and the influence of broadening

For fitting superconducting gap spectra and extracting Δ, we used two different BCS-based fitting models.

1) The Maki equation for the density of states (*45*), based on Pauli paramagnetism in small superconductors:

$$\rho(E) = \Re\left(\frac{u}{\sqrt{u^2-1}}\right),$$

where $u = \frac{E}{\Delta} + \zeta \frac{u}{\sqrt{1-u^2}}$ (see ref. (*64*) for the analytical solution) and ζ describes the pair-breaking parameter. Note that this description is valid in absence of magnetic field, i.e. no spin-splitting.

2) The Dynes equation (*50*), a phenomenological equation to capture the broadening due to finite quasiparticle recombination times:

$$\rho(E) = \frac{E - i\Gamma}{\sqrt{(E - i\Gamma)^2 - \Delta^2}} \, .$$

The Maki equation renormalizes the coherence peak height, while the Dynes equation induces a Gaussian type of broadening which may induce in-gap conductance.

In Fig. S5, we plot a typical superconducting gap of a film with coverage 8.5 ML (blue circles), and in Fig. S5A fitted with the Maki equation (red line), with a magnified focus of the fit on the left coherence peak. We find an excellent agreement between data and fit for Δ = 0.46 meV, ζ = 0.02 meV if we include a Fermi-Dirac broadening with an effective temperature T_{eff} = 250 mK. Noteworthy, this approach does not specify the origin of the broadening effects and can include broadening contributions that are unrelated to temperature. Moreover, this effective temperature is considerably larger than the measurement temperature of T = 30 mK, which suggests additional non-thermal broadening. Previously, we calibrated the energy resolution of our experimental setup at ~65 µeV in ref. (*65*). Here, we consider three potential broadening contributions.

Firstly, tunneling spectroscopy at mK temperature suffers from an intrinsic broadening mechanism, based on the quantization of the tunneling current and its interaction with the electromagnetic environment (*66*). The capacitive noise can be described by the $P(E)$ theory and in practice adds a Gaussian broadening $P_N(E) = \frac{1}{\sqrt{4\pi E_C k_B T}} \exp\left[-\frac{E^2}{4E_C k_B T}\right]$, where $E_C = Q^2/2C_J$ is the charging energy of the Cooper pairs with Q = 2e and C_J the capacitance of the tunnel junction. In Fig. S5B, we fitted the same spectrum with the Maki equation, extended by the $P_N(E)$ contribution and find that we require a capacitance of C_J = 0.25 fF to account for the broadening, if T_{eff} equals the measurement temperature. However, assuming the proposed model in ref. (*66*) for a tip with diameter d = 0.3 mm and an opening angle of α = 60°, we find a capacitance of 12.7 fF instead, suggesting that the $P_N(E)$ broadening is not the main broadening contribution.

Secondly, we considered a sample with a convolution of two-layer thicknesses, where each layer has a distinct value of Δ, namely Δ_N and Δ_{N+1}. We model this by combining two Maki equations for each value of Δ, considering a 50% contribution of each Δ. In Fig. S5C, we illustrate the resultant convolution as well as a decomposition of each of gap contributions (dashed lines). The result shows a good match with the data, using T_{eff} = 150 mK and a difference $|\Delta_N - \Delta_{N+1}|$ = 70 µeV. Based on the data shown in Fig. 2B it is reasonable to consider such differences in the limit where N < 10 ML. However, we also observed that the inherent broadening remained for higher coverages, and therefore we cannot conclude that this is solely responsible for the observed gap broadening. Ultimately, a gap difference larger than the energy resolution is required to confirm the existence of such a double-gap proximity effect, as this would result in a kink in the spectral shape.

Lastly, we considered the hybridization model proposed in ref. (*49*). For Pb films on black phosphorus (BP), weak hybridization between the bands of BP and the quantum well states of Pb leads to a non-thermal broadening of the superconducting gap and anisotropic vortices. However, for Al films on Si, no renormalization of the superconducting gap is not expected, as indicated by the observed isotropic vortices. A good agreement between the model and the data (Fig. S5D) is only found by taking a large amount of anisotropy (e.g. $m_x/m_y = 8$, where m_x and m_y describe the effective mass of the Al band in *x*- and *y*-direction respectively) and a weighting function (using T_{eff} = 150 mK). However, such large anisotropy values are expected to influence the vortex shape, as seen for vortices in Pb on black phosphorus, but the vortices in Al are isotropic (Fig. 3B, Fig. S9). Therefore, we expect that the broadening mechanism in Al films does not stem from anisotropic hybridization effects.

S3 Temperature calibration for temperature dependent measurements above 1 K

To perform temperature dependent measurements, we heated the sample with a Zener diode. To calibrate the temperature sensor, we measured the temperature dependence of V(111) bulk, and a thick Sn film epitaxially grown on Si(111)-(7x7) (*67-71*). We prepared the V(111) sample with repeated

sputter and anneal cycles (T_{anneal} = 850 °C). For the second study, we deposited Sn on a clean Si(111)-(7x7) substrate held at T ~ 110 K . Based on a previous calibration, we expected a coverage between 120-150 ML, i.e. in the bulk regime.

In Fig. S6, we plot the dI/dV spectroscopy measured as a function of temperature, where the temperature value refers to the sensor reading, for the two aforementioned samples. We extracted Δ(T) by fitting a Dynes equation (black lines). The resultant Δ(T) is plotted below each subfigure. Both material systems show an excellent agreement with the BCS equation and the measured Δ(T). Using the extracted values for Δ and T_c ($\Delta_{\mathrm{Sn}}^{T=0}$= 0.67 ± 0.03 meV, $T_{c,\mathrm{Sn}}$ = 3.9 ± 0.2 K, $\Delta_{\mathrm{V}}^{T=0}$ = 0.79 ± 0.04 meV, $T_{c,\mathrm{V}}$ = 5.1 ± 0.2 K), we find that the BCS ratio $2\Delta/k_BT_c$ = 3.99 ± 0.27 for Sn/Si(111) and $2\Delta/k_BT_c$ = 3.60 ± 0.23 for V(111). Since these values, in particular those for V, match with literature (*67, 70, 71*), we conclude that the temperature of the STM sensor gives a reliable measurement of the sample temperature in the range between 1.2 K and 5.1 K.

Temperature dependence of three thin Al films

Temperature dependent measurements as described in the main manuscript were performed for three other Al films with coverages of 6.3 ML, 5.3 ML and 4.2 ML, as shown in Fig. S7A-C. We apply the same analysis and extract $\Delta_{6.3\,\mathrm{ML}}^{T=0}$= 0.39 ± 0.02 meV, $\Delta_{5.3\,\mathrm{ML}}^{T=0}$= 0.43 ± 0.02 meV and $\Delta_{4.2\,\mathrm{ML}}^{T=0}$= 0.51 ± 0.02 meV, as well as $T_{c,6.3\,\mathrm{ML}}$ = 2.51 ± 0.10 K, $T_{c,5.3\,\mathrm{ML}}$ = 2.90 ± 0.11 K and $T_{c,4.2\,\mathrm{ML}}$ = 3.15 ± 0.08 K. Here, the error bars represent the standard deviation given by the BCS fit.

S4 Effect of minimal annealing on the film morphology and resultant superconductivity

In order to explore the role of room temperature annealing on the morphology and resultant superconducting gap, we prepared an additional sample with a coverage of 8.5 ML. After cold deposition, the sample was minimally annealed at room temperature (~ 1 min). The resulting sample shows small islands on top of a closed layer with atomic resolution visible, as shown in Fig. S8A. The moiré-like periodicity was not visible, likely obstructed by the abundance of small islands. Still, we find a spatially homogeneous gap with a gap size of ~ 0.5 meV, shown in Fig. S8B, which is similarly enhanced compared to the annealed sample with 8.5 ML coverage (see Fig. 2A).

S5 Al films in (high) magnetic fields

Theoretical model for vortex simulations

To model the superconducting vortices, we use quasiclassical Keldysh theory, which is valid when the Fermi energy is much larger than all other energy scales. Assuming that the mean free path is also much less than the coherence length, the system can be fully described by the momentum-averaged quasiclassical Green's function,

$$\check{g} = \begin{pmatrix} \hat{g}^R & \hat{g}^K \\ 0 & \hat{g}^A \end{pmatrix}, \tag{1}$$

where $\hat{g}^R$, $\hat{g}^A$ and $\hat{g}^K$ are the retarded, advanced and Keldysh components of the Green's function, respectively. In thermal equilibrium it is sufficient to find the retarded Green's function, since $\hat{g}^A = -\hat{\tau}_z(\hat{g}^R)^\dagger\hat{\tau}_z$ and $\hat{g}^K = (\hat{g}^R - \hat{g}^A)\tanh(\beta\varepsilon/2)$, where $\hat{\tau}_z$ = diag(1,1,-1,-1), β is the inverse temperature and ε is the energy.

The retarded quasiclassical Green's function solves the Usadel equation (*58*),

$$D\tilde{\nabla} \cdot \left(\hat{g}^R\tilde{\nabla}\hat{g}^R\right) + i\left[\hat{\tau}_z(\varepsilon + i\Gamma) + h^{\parallel}\hat{\sigma}_z + \hat{\Delta}, \hat{g}^R\right] = 0, \tag{2}$$

Here, D is the diffusion constant, Γ is the Dynes parameter, $h^{\parallel}$ is the spin-splitting field, $\hat{\Delta}$ = antidiag(Δ,-Δ,Δ*,-Δ*) and the covariant derivative is

$$\tilde{\boldsymbol{\nabla}}\hat{g}^R = \boldsymbol{\nabla}\hat{g}^R - ie[\hat{\tau}_z\boldsymbol{A}, \hat{g}^R],$$

(3)

where e = -|e| is the electron charge and $\boldsymbol{A}$ is the vector potential. The superconducting gap parameter, Δ, must solve the gap equation (*72*).

$$\Delta = \frac{1}{16\log(2\omega_D/\Delta_0)}\int_{-\omega_D}^{\omega_D} d\varepsilon\, \mathrm{Tr}\left[-i\hat{\sigma}_y(\hat{\tau}_x - i\hat{\tau}_y)\hat{g}^K\right],$$

(4)

where ω_D is the Debye frequency and Δ_0 is the zero-temperature BCS bulk solution. Additionally, the vector potential must solve Maxwell's equation. Assuming $\boldsymbol{\nabla}\cdot\boldsymbol{A} = 0$, Maxwell's equation reads (*57*)

$$\boldsymbol{\nabla}^2\boldsymbol{A} = -\frac{\mu N_0 eD}{4}\int_{-E_c}^{E_c} d\varepsilon\, \mathrm{Tr}\left[\hat{\tau}_z\left(\check{g}\tilde{\boldsymbol{\nabla}}\check{g}\right)^K\right],$$

(5)

where $\boldsymbol{\nabla}^2$ is the vector Laplacian, E_c is a cut-off energy, μ is the magnetic permeability and N_0 is the normal state density of states at the Fermi level. For a self-consistent solution, we must solve eqs. (2), (4) and (5) simultaneously.

To model the vortex, we solve eqs. (2), (4) and (5) on an infinite plane with a single phase winding in the gap parameter around the origin and assume that the vector potential points in the azimuthal direction. That is, using polar coordinates, $\Delta(\boldsymbol{r}) = \Delta(r)e^{i\theta}$ and $\boldsymbol{A} = A\boldsymbol{e}_\theta$, where $\boldsymbol{e}_\theta$ is the unit vector in the θ-direction. The latter means that $\boldsymbol{\nabla}^2\boldsymbol{A} = (\nabla^2 A - A/r^2)\boldsymbol{e}_\theta$. We solve the equations numerically by using the Ricatti parametrization,

$$\hat{g}^R = \begin{pmatrix} N & 0 \\ 0 & -\tilde{N}\end{pmatrix}\begin{pmatrix} 1+\gamma\tilde{\gamma} & 2\gamma \\ 2\tilde{\gamma} & 1+\tilde{\gamma}\gamma\end{pmatrix},$$

(6)

where $\tilde{\gamma}(\epsilon) = \gamma^*(-\epsilon)$ and $N = (1-\gamma\tilde{\gamma})^{-1}$. If we choose the spin-quantization axis to be along the in-plane magnetic field, we need only solve for two components of γ, and we may write

$$\gamma(\boldsymbol{r},\theta) = \begin{pmatrix} 0 & \gamma_1(r) \\ -\gamma_2(r) & 0\end{pmatrix}e^{i\theta}.$$

(7)

Next, we make all quantities dimensionless by dividing eqs. (2) and (4) by the superconducting gap at $r \to \infty$, Δ^∞, and multiplying eq. (5) by $2e\xi$, where $\xi = \sqrt{D/\Delta^\infty}$ is the diffusive superconducting coherence length. We define the dimensionless quantities $\bar{\varepsilon} = \varepsilon/\Delta^\infty$, $\bar{h}^{\parallel} = h^{\parallel}/\Delta^\infty$, $\bar{\Gamma} = \Gamma/\Delta^\infty$, $\bar{\Delta} = \Delta/\Delta^\infty$, $\bar{r} = r/\xi$, and $\bar{A} = 2e\xi A$. To solve the equations numerically on an infinite domain, we also define $z = \bar{r}/(1+\bar{r})$.

From eq. (2) we find that

$$(1-z)^4\frac{\partial^2\gamma_{1/2}}{\partial z^2}+\frac{(1-2z)(1-z)^3}{z}\frac{\partial\gamma_{1/2}}{\partial z}-\frac{(1-z)^2}{z^2}\gamma_{1/2}+2i\left(\bar{\varepsilon}+i\bar{\Gamma}\pm\bar{h}^{\parallel}\right)\gamma_{1/2}-i\bar{\Delta}-i\bar{\Delta}^*\gamma_{1/2}^2$$
$$+\frac{2\tilde{\gamma}_{1/2}}{1+\gamma_{1/2}\tilde{\gamma}_{2/1}}\left[\frac{(1-z)^2\gamma_{1/2}^2}{z^2}-(1-z^4)\left(\frac{\partial\gamma_{1/2}}{\partial z}\right)^2\right]$$
$$-\bar{A}\gamma_{1/2}\frac{1-\gamma_{1/2}\tilde{\gamma}_{2/1}}{1+\gamma_{1/2}\tilde{\gamma}_{2/1}}\left(\bar{A}-\frac{2(1-z)}{z}\right)=0,$$

(8)

from eq. (4), we get

$$\bar{\Delta}=\frac{1}{2\log(2\omega_D/\Delta_0)}\int_{-\omega_D/\Delta^\infty}^{\omega_D/\Delta^\infty}\mathrm{d}\bar{\varepsilon}\,\Re\left(\frac{\gamma_1}{1+\gamma_1\tilde{\gamma}_2}+\frac{\gamma_2}{1+\gamma_2\tilde{\gamma}_1}\right)\tanh\left(\frac{\beta\varepsilon}{2}\right),$$

(9)

and from eq. (5), we get that

$$(1-z)^4\frac{\partial^2\bar{A}}{\partial z^2}-\frac{(1-z)^2}{z^2}\bar{A}+\frac{(1-2z)(1-z)^3}{z}\frac{\partial\bar{A}}{\partial z}$$
$$=\frac{1}{\kappa^2}\left(\bar{A}-\frac{1-z}{z}\right)\int_0^{E_c/\Delta^\infty}\mathrm{d}\bar{\varepsilon}\,\Im\left(\frac{\gamma_1\tilde{\gamma}_2}{\left(1+\gamma_1\tilde{\gamma}_2\right)^2}+\frac{\gamma_2\tilde{\gamma}_1}{\left(1+\gamma_2\tilde{\gamma}_1\right)^2}\right)\tanh\left(\frac{\beta\varepsilon}{2}\right),$$

(10)

where κ is a dimensionless parameter which we set equal to 5 in all calculations.

Having found the quasiclassical Green's function, one can calculate the local density of states, and thereby the theoretical prediction for the current as measured by the STM. In terms of the Ricatti parameters, the local density of states reads

$$\rho(E)=\frac{N_0}{2}\Re\left(\frac{1-\gamma_1\tilde{\gamma}_2}{1+\gamma_1\tilde{\gamma}_2}+\frac{1-\gamma_2\tilde{\gamma}_1}{1+\gamma_2\tilde{\gamma}_1}\right).$$

(11)

Assuming a constant tunnelling transmission, we get that the differential conductance is

$$\frac{\mathrm{d}I}{\mathrm{d}V}=C\int_{-\infty}^{\infty}\mathrm{d}\bar{\varepsilon}\;\Re\left(\frac{1-\gamma_1\tilde{\gamma}_2}{1+\gamma_1\tilde{\gamma}_2}+\frac{1-\gamma_2\tilde{\gamma}_1}{1+\gamma_2\tilde{\gamma}_1}\right)\frac{\exp(\varepsilon-eV/k_\mathrm{B}T_\mathrm{eff})}{(1+\exp(\varepsilon-eV/k_\mathrm{B}T_\mathrm{eff}))^2},$$

(12)

where V is the applied bias voltage, k_B is the Boltzmann constant and T_{eff} is an experimental broadening parameter and C is a proportionality constant.

The presence of the spin-splitting field $h^{\parallel}$ induces odd-frequency superconducting correlations. These correlations are characterized by $\left|\mathrm{Tr}\left[\hat{\sigma}_x\left(\hat{\tau}_x-i\hat{\tau}_y\right)\hat{g}^K\right]\right|>0$. However, unlike the even frequency correlations, the odd-frequency correlations vanish upon integration over $\bar{\varepsilon}$ and therefore require another correlation function compared to eq. (4). Here we use the correlation function obtained by multiplying the integrand with $\bar{\varepsilon}$,

$$\Psi_\mathrm{odd}=\frac{-i}{16}\int_{-\omega_D/\Delta^\infty}^{\omega_D/\Delta^\infty}\mathrm{d}\bar{\varepsilon}\,\mathrm{Tr}\left[\hat{\sigma}_x\left(\hat{\tau}_x-i\hat{\tau}_y\right)\hat{g}^K\right]\bar{\varepsilon}=\int_{-\omega_D/\Delta^\infty}^{\omega_D/\Delta^\infty}\mathrm{d}\bar{\varepsilon}\,\Im\left(\frac{\gamma_1}{1+\gamma_1\tilde{\gamma}_2}-\frac{\gamma_2}{1+\gamma_2\tilde{\gamma}_1}\right)\tanh\left(\frac{\beta\varepsilon}{2}\right)\bar{\varepsilon}.$$

(13)

This is equivalent to differentiating the anomalous Green's function with respect to relative time (*73*).

When calculating the electric current density, we can separate the contributions from the even-frequency correlations and the odd-frequency correlations. In terms of the even-frequency retarded anomalous Green's function,

$$f_{\text{even}} = \frac{\gamma_1}{1+\gamma_1\tilde{\gamma}_2} + \frac{\gamma_2}{1+\gamma_2\tilde{\gamma}_1}, \tag{14}$$

and the odd-frequency retarded anomalous Green's function,

$$f_{\text{odd}} = \frac{\gamma_1}{1+\gamma_1\tilde{\gamma}_2} - \frac{\gamma_2}{1+\gamma_2\tilde{\gamma}_1}, \tag{15}$$

the electric supercurrent density can be written

$$j_s = j_{s,\text{even}} + j_{s,\text{odd}}, \tag{16}$$

where

$$j_{s,\text{even}} = -N_0 eD\left(2eA - \frac{1}{r}\right)\int_{-E_c}^{E_c} \mathrm{d}\varepsilon\, \Im\left(f_{\text{even}}\tilde{f}_{\text{even}}\right)\tanh\left(\frac{\beta\varepsilon}{2}\right), \tag{17}$$

is the electric current density associated with the even-frequency correlations and

$$j_{s,\text{odd}} = N_0 eD\left(2eA - \frac{1}{r}\right)\int_{-E_c}^{E_c} \mathrm{d}\varepsilon\, \Im\left(f_{\text{odd}}\tilde{f}_{\text{odd}}\right)\tanh\left(\frac{\beta\varepsilon}{2}\right), \tag{18}$$

is the electric current density associated with the odd-frequency correlations.

Abrikosov vortex lattice of thin film Al

In order to compare the typical Abrikosov vortex with the MTF vortex structure, we measured a zero-bias conductance map in the same area as the map shown in Fig. 4A, but now with an applied magnetic field of $B^{\perp}$ = 30 mT and $B^{\parallel}$ = 0 T (Fig. S9A). The map shows several round vortices, which are likely influenced by the tip during scanning (fast scan direction: horizontal). We characterize the Abrikosov vortex structure by taking spectra along a horizontal line, plotted in Fig. S9B. We observe that the spectral gap reduces gradually towards the vortex core and can reproduce this trend by solving the self-consistent gap equation in the absence of in-plane fields (Fig. S9C), while keeping the other parameters the same as in Fig. 4E. The zero-bias conductance profile, as plotted in Fig. 4B, shows a much sharper peak, compared to the MTF vortex.

In-plane magnetic field dependence on three thin Al films

The measurement in Fig. 3C is repeated on two additional films with coverages of 11.7 ML, and 5.6 ML, as shown in Fig. S10A-B. Additionally, we plot the extracted gap values as a function of $B^{\parallel}$ in Fig. S10C, for each of the four film coverages presented in Fig. 3C and Fig. S10A-B. We find that the gap values are robust against $B^{\parallel}$ fields, demonstrating that the MTF effect can be understood as the Zeeman shifting of two spin-polarized gap functions, without a modification of the gap value. We note that the presented spectrum at $B^{\parallel}$ = 4.0 T in Fig. S10B is likely influenced by a nearby MTF vortex.

Zero-bias conductance maps in in-plane and vector magnetic fields

To ascertain the origin of the vortex structure in Fig. 6, we imaged the zero-bias conductance in large in-plane magnetic field ($B^{\parallel}$= 4 T) for a 5.6 ML Al film in an 800 nm x 500 nm image (Fig. S11A/B). By measuring the density of vortices, we can estimate the number of flux lines that penetrate the film due to an out-of-plane component. While the sparsity of objects is low in Fig. S11B, we estimate that the intervortex distance *d* is between 400 – 450 nm. Using $B^{\mathrm{tilt}} = \sqrt{4/3}\,\Phi_0/d^2$, we find that the corresponding out-of-plane component is B^{tilt} ~ 12 to 15 mT. The angle between the applied field ($B^{\parallel}$ = 4 T) and the sample is therefore α_{tilt} ~ 0.2°. We suspect that the placement of the Si wafer on the sample plate is the main contribution to this angle, for this particular sample. By measuring the zero-bias conductance for the same area in a vector magnetic field of $B^{\parallel}$ = 3 T and $B^{\perp}$ = 30 mT, we expect a total out-of-plane component of either ~40 mT ($B^{\perp}$ + B^{tilt}) or ~20 mT ($B^{\perp}$ - B^{tilt}), depending on the orientation of the tilt. From the flux density in Fig. S11C, we conclude that $B^{\perp}$ and B^{tilt} align, resulting in a higher vortex density. Comparable tilt angles are expected for all samples in this study.

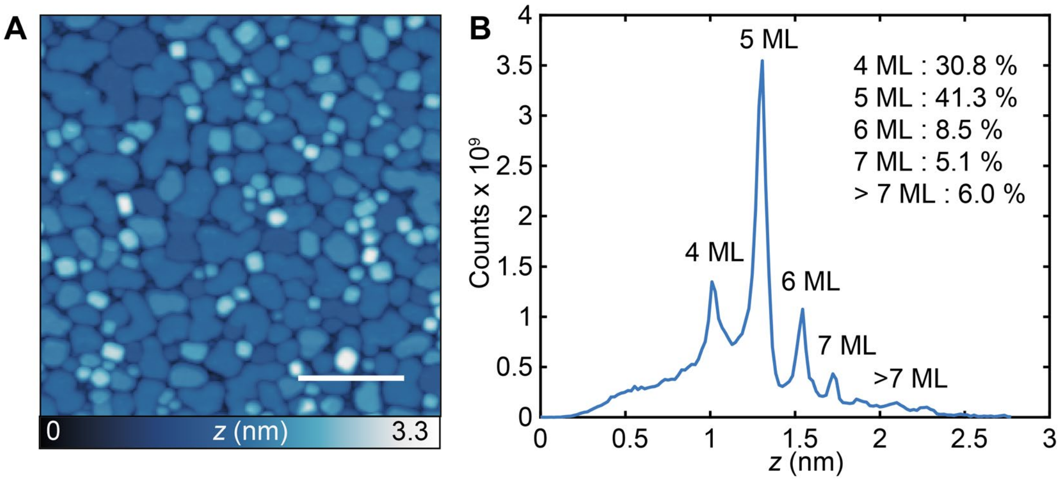

Fig. S1. Coverage calibration with room-temperature growth. (A) Constant-current image of Al islands grown on Si(111)-(7x7) after depositing Al for 12 minutes (V_s = 1 V, I_t = 10 pA, scale bar = 50 nm, $T \approx 7$ K). (B) Apparent height histogram of (A) with layer numbers assigned. Inset numbers: percentages for each layer thickness as extracted by flooding analysis.

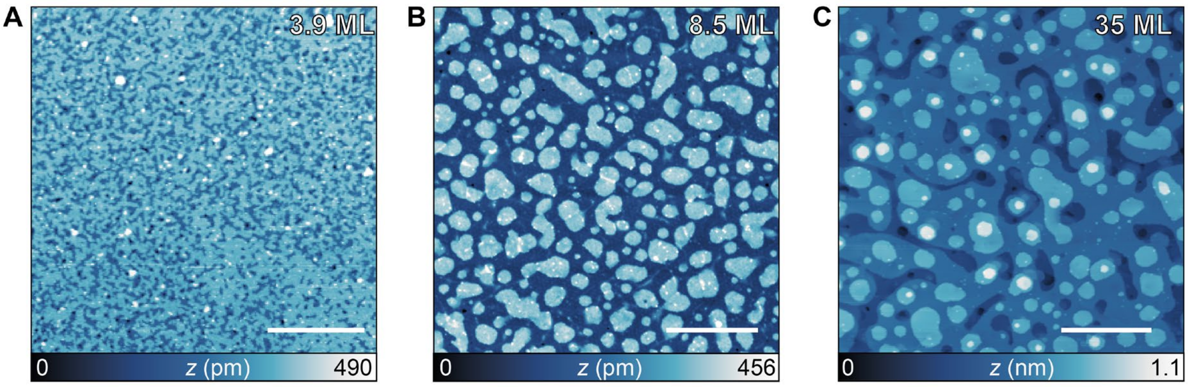

Fig. S2. Morphology of various coverages. Constant-current STM images of Al films with (A) 3.9 ML, (B) 8.5 ML, and (C) 35.1 ML coverages (V_s = 1 V, I_t = 10 pA, scale bar = 50 nm, (A,B) $T \approx 30$ mK and (C) $T \approx 7$ K).

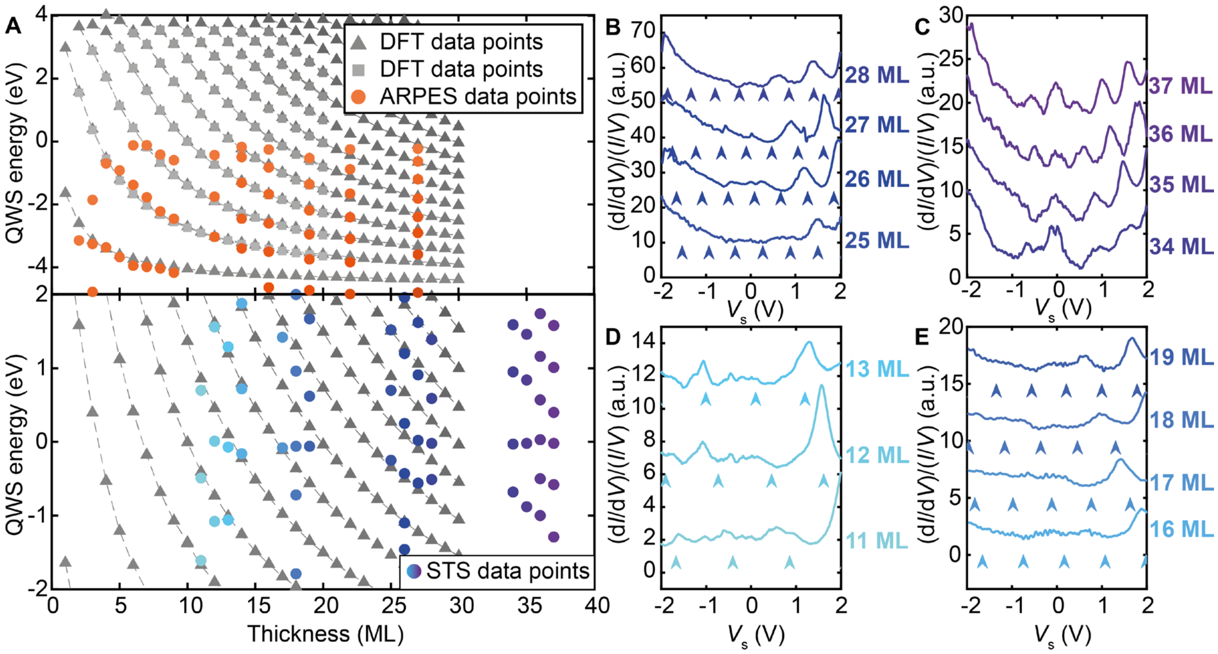

Fig. S3. d*I*/d*V* comparison to QWS derived from DFT and ARPES. (A) Calculated QWS energies from DFT (gray), extracted from ref. (*42, 43*), and ARPES peak positions (orange), extracted by eye from ref. (*44*), for Al(111) films on Si(111) as a function of film thickness. (B-E) d*I*/d*V* spectra normalized by the total conductance *I*/*V*, obtained for four Al films with coverages of (B) 26.3 ML, (C) 35.1 ML, (D) 11.7 ML and (E) 17.4 ML, where the arrows indicate the DFT energies in (A) (stabilized V_s = 2 V, I_t = 200 pA or 500 pA, V_{mod} = 5 mV, (B,C,E) $T \approx 30$ mK and (D) $T \approx 7$ K).

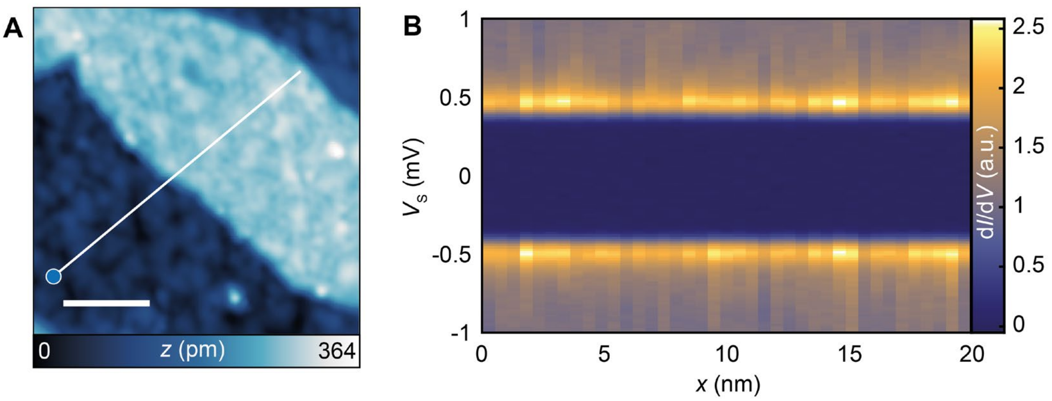

Fig. S4. Spatial measurement of superconductivity. (A) Constant-current STM image of an Al film with 8.5 ML coverage (V_s = 100 mV, I_t = 10 pA, scale bar = 5 nm). (B) dI/dV spectra along a line across 8 ML and 9 ML regions and across the long-range periodicity (line indicated in (A); each spectrum is stabilized at V_s = 3 mV, I_t = 200 pA, V_{mod} = 20 μV).

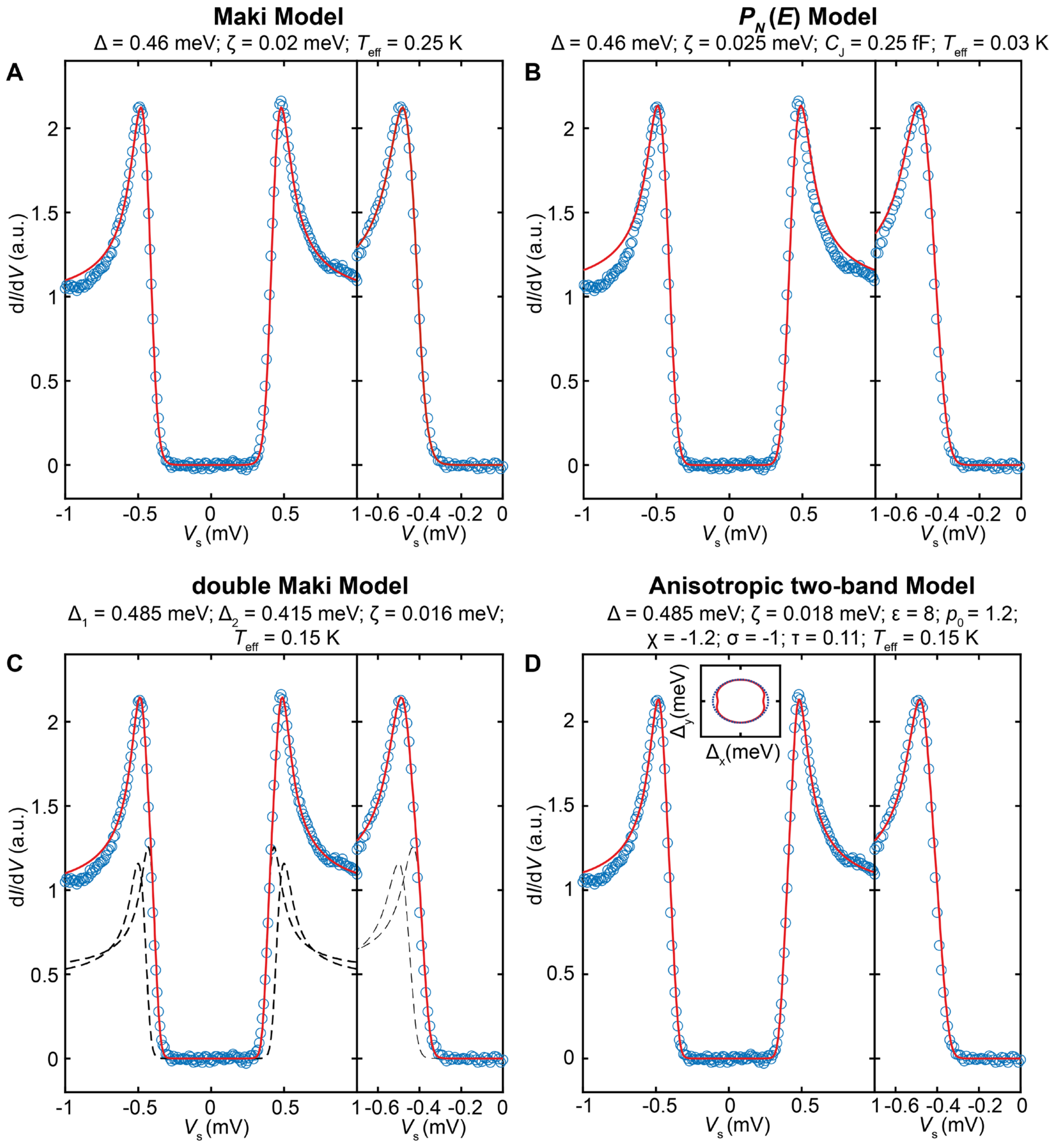

Fig. S5. Comparison of different fitting methods and broadening contributions. Example of a typical superconducting gap spectrum (blue dots) measured on an Al film with 8.5 ML coverage, compared to different models (red lines) (stabilized at V_s = 3 mV, I_t = 200 pA, V_{mod} = 20 µV). (A) Fit with the Maki equation, (B) a $P_N(E)$ broadening Maki equation, (C) a double Maki equation using two different gap sizes Δ_1 and Δ_2 (individual contributions in black dashed lines), and (D) a Maki-based anisotropic two-band model. The fitting parameters, indicated above each plot, are the gap size Δ, the Maki broadening ζ, the effective temperature T_{eff}, the junction capacitance C_J, the degree of anisotropy $\varepsilon = \frac{m_x^*}{m_y^*} - 1$ and the effective mass m_i^* of the Al band in direction *i*, $p_0 = \tau/\mu$, $\sigma = \delta/\mu$, $\chi = m^*/m_x^*$, τ is the coupling between bands, μ is the chemical potential, and δ is the energy offset between bands. The inset of (D) shows the resulting anisotropic gap structure.

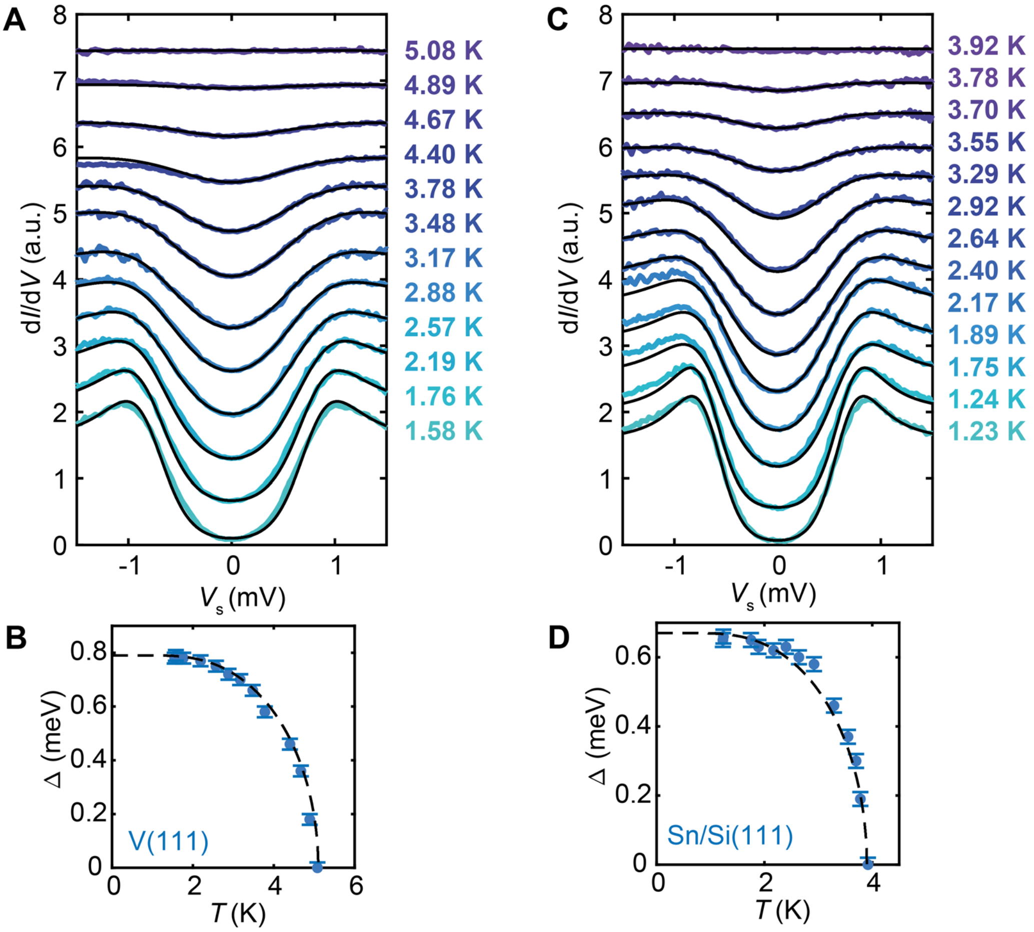

Fig. S6. Temperature calibration of the temperature dependent measurements made above 1 K. (A) Superconducting gap measurements as a function of STM temperature (see legend; artificially offset) on bulk V(111) (stabilized at V_s = 5 mV, I_t = 300 pA, V_{mod} = 100 µV). (B) Extracted Δ(T) fitted with the BCS equation (dashed line). (C) Superconducting gap measurements as a function of STM temperature (artificially offset) for a Sn film with an estimated coverage of 120-150 ML, grown on Si(111) (stabilized at V_s = 5 mV, I_t = 300 pA, V_{mod} = 100 µV). (D) Extracted Δ(T) fitted with the BCS equation (dashed line).

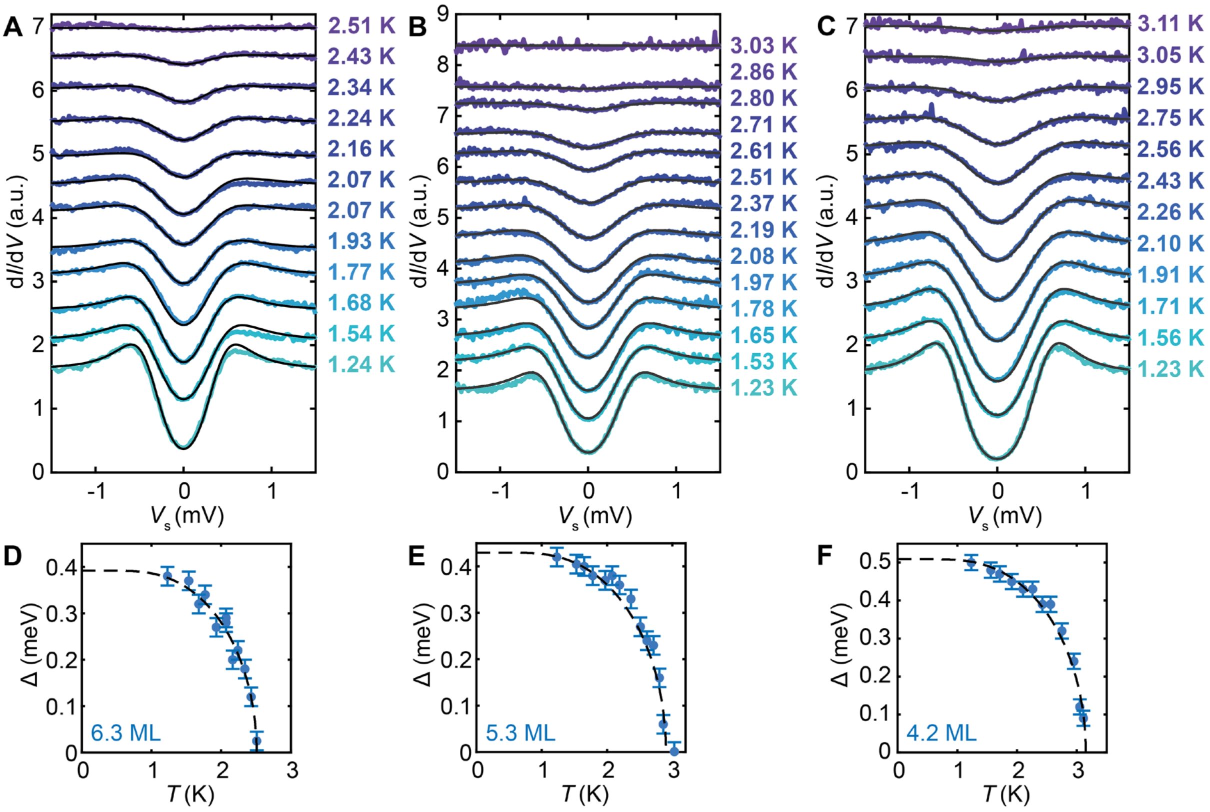

Fig. S7. Temperature dependence of three Al films. (A-C) Temperature dependent spectra (artificially offset) fitted with the Dynes equation (stabilized at V_s = 5 mV, I_t = 300 pA, V_{mod} = 100 μV). (D-F) Extracted $\Delta(T)$ fitted with the BCS equation (dashed lines). The film coverages of (A,D) 6.3 ML, (B,E) 5.3 ML and (C,F) 4.2 ML yield a BCS ratio of 3.63 ± 0.02, 3.44 ± 0.02 and 3.75 ± 0.01 respectively (see Fig. 2E).

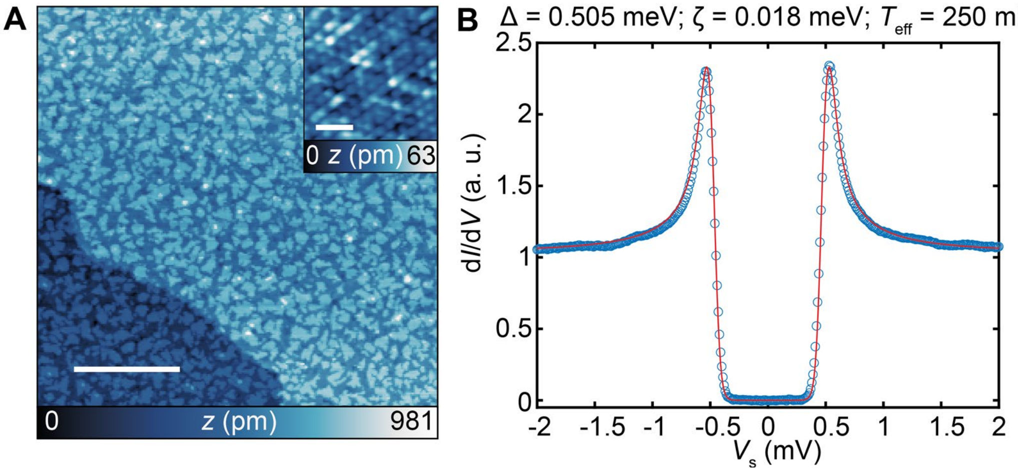

Fig. S8. Morphology and superconductivity after minimal annealing. (A) Constant-current image of the morphology of an 8.5 ML Al film after cold-growth and minimal annealing (transfer time of ~ 1 minute; V_s = 2 V, I_t = 10 pA, scale bar = 50 nm, $T \approx 7$ K). Inset: Constant-current STM image showing atomic resolution in a flat region (V_s = 3 mV, I_t = 500 pA, scale bar = 1 nm). (B) Spatially averaged superconducting gap fitted with the Maki equation (red line; parameters indicated above graph; stabilized at V_s = 3 mV, I_t = 200 pA, V_{mod} = 20 μV).

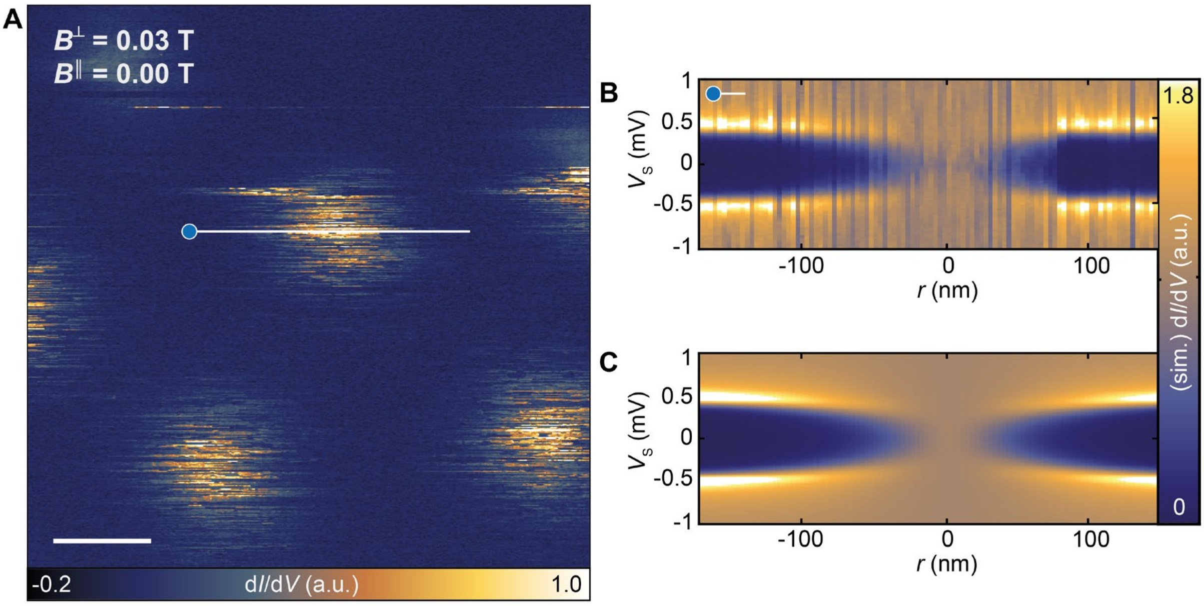

Fig. S9. Abrikosov vortices. (A) Constant-contour dI/dV map with $B^{\perp}$ = 30 mT ($B^{\parallel}$ = 0.0 T) for an 8.5 ML film (height recorded at V_s = 10 mV, I_t = 10 pA; image taken with V_s = 0 mV and z-offset = 100 pm, V_{mod} = 50 µV, scale bar = 100 nm). (B) dI/dV spectra along a horizontal line across a vortex core (stabilized at V_s = 3 mV, I_t = 200 pA, V_{mod} = 20 µV). (C) Simulated dI/dV signal by solving the self-consistent gap equation using $h^{\parallel}/\Delta^{\infty}$ = 0, ξ = 42 nm, Γ = 0.001 Δ^{∞}, κ = 5, and broadened with T_{eff} = 250 mK.

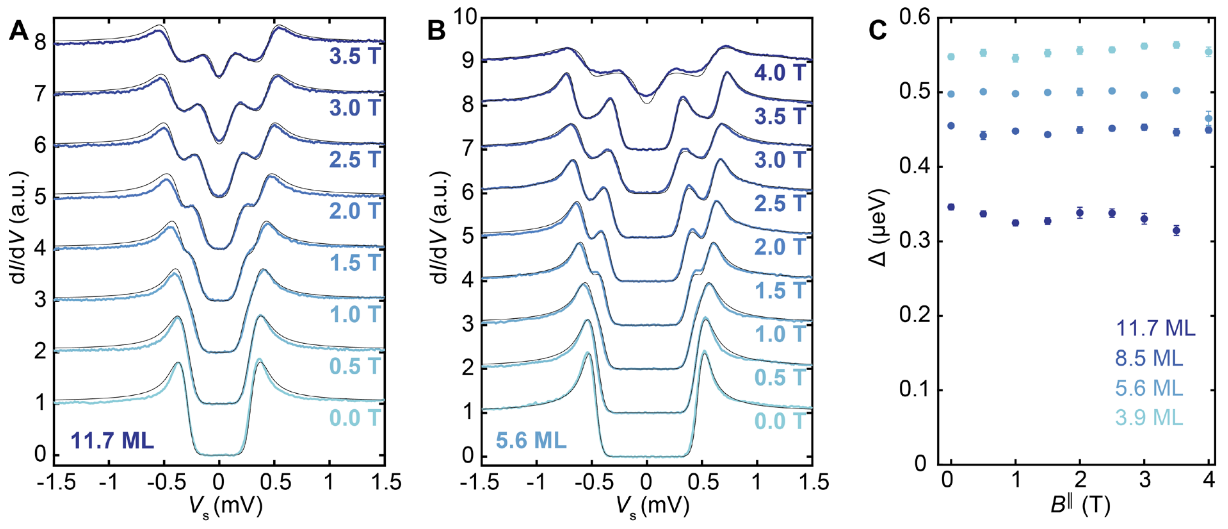

Fig. S10. In-plane magnetic field dependence for two additional Al films. Evolution of the SC gap in in-plane magnetic field $B^{\parallel}$ for film coverages of (A) 11.7 ML, and (B) 5.6 ML. Black lines are fits using a double-Maki fit with Zeeman splitting (stabilized at V_s = 3 mV, I_t = 200 pA, V_{mod} = 20 μV) (C) Extracted Δ for four Al coverages (also see Fig. 3C) as a function of $B^{\parallel}$. Note that Δ stays approximately constant while the two spin-polarized gaps shift with respect to each other by ΔE_Z (see Fig. 3D)

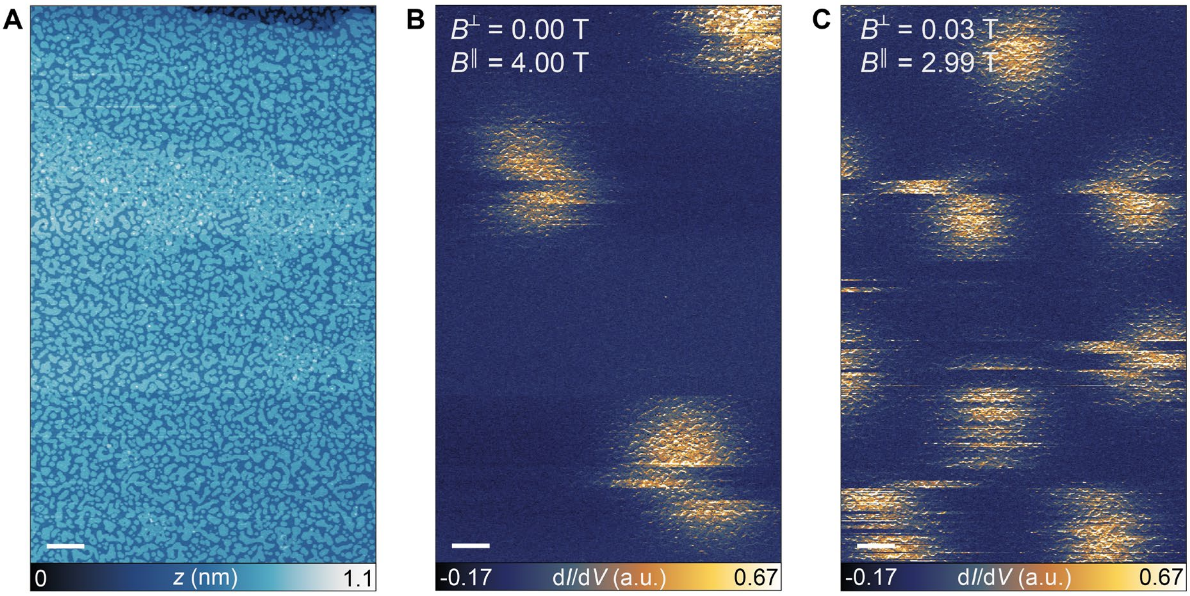

Fig. S11. Zero-bias conductance in large in-plane and vector fields. (A) Constant-current STM image of a 5.6 ML Al film and (B) simultaneously recorded constant-contour d*I*/d*V* map with $B^{\parallel}$ = 4 T ($B^{\perp}$ = 0 mT). (C) Constant-contour d*I*/d*V* map with $B^{\perp}$ = 30 mT and $B^{\parallel}$ = 2.99 T (height profiles recorded at V_s = 10 mV, I_t = 10 pA; d*I*/d*V* maps taken with V_s = 0 mV and *z*-offset = 100 pm, V_{mod} = 50 μV, scale bar = 50 nm).
